# Coronary calcium score and emphysema extent on different CT radiation dose protocols in lung cancer screening

**DOI:** 10.1007/s00330-024-11254-w

**Published:** 2024-12-20

**Authors:** Roberta Eufrasia Ledda, Gianluca Milanese, Maurizio Balbi, Federica Sabia, Camilla Valsecchi, Margherita Ruggirello, Andrea Ciuni, Giulia Tringali, Nicola Sverzellati, Alfonso Vittorio Marchianò, Ugo Pastorino

**Affiliations:** 1 Thoracic Surgery Unit, Fondazione IRCCS Istituto Nazionale Tumori, Milan, Italy; 2 Department of Medicine and Surgery (DiMeC), University of Parma, Parma, Italy; 3 Radiology Unit, San Luigi Gonzaga Hospital, Department of Oncology, University of Turin, Orbassano (TO), Italy; 4 Division of Radiology, Fondazione IRCCS Istituto Nazionale dei Tumori, Milan, Italy; 5 Radiological Sciences Unit, University Hospital of Parma, Parma, Italy

**Keywords:** Ultra-low-dose computed tomography, Coronary artery calcification, Pulmonary emphysema, Lung cancer screening, Automated quantification

## Abstract

**Objectives:**

To assess the consistency of automated measurements of coronary artery calcification (CAC) burden and emphysema extent on computed tomography (CT) images acquired with different radiation dose protocols in a lung cancer screening (LCS) population.

**Materials and methods:**

The patient cohort comprised 361 consecutive screenees who underwent a low-dose CT (LDCT) scan and an ultra-low-dose CT (ULDCT) scan at an incident screening round. Exclusion criteria for CAC measurements were software failure and previous history of CVD, including coronary stenting, whereas for emphysema assessment, software failure only. CT images were retrospectively analyzed by a fully automated AI software for CAC scoring, using three predefined Agatston score categories (0–99, 100–399, and ≥ 400), and emphysema quantification, using the percentage of low attenuation areas (%LAA). Demographic and clinical data were obtained from the written questionnaire completed by each participant at the first visit. Agreement for CAC and %LAA categories was measured by the k-Cohen Index with Fleiss-Cohen weights (K_w_) and Intraclass Correlation Coefficient (ICC) with 95% Confidence Interval (CI).

**Results:**

An overlap of CAC strata was observed in 275/327 (84%) volunteers, with an almost perfect agreement (K_w_ = 0.86, 95% CI 0.82–0.90; ICC = 0.86, 95% CI 0.79–0.90), while an overlap of %LAA strata was found in 204/356 (57%) volunteers, with a moderate agreement (K_w_ = 0.57, 95% CI 0.51–0.63; ICC = 0.57, 95% CI 0.21–0.75).

**Conclusion:**

Automated CAC quantification on ULDCT seems feasible, showing similar results to those obtained on LDCT, while the quantification of emphysema tended to be overestimated on ULDCT images.

**Key Points:**

***Question*** *Evidence demonstrating that coronary artery calcification and emphysema can be automatedly quantified on ultra-low-dose chest CT is still awaited.*

***Findings*** *Coronary artery calcification and emphysema measurements were similar among different CT radiation dose protocols; their automated quantification is feasible on ultra-low-dose CT.*

***Clinical relevance*** *Ultra-low-dose CT-based LCS might offer an opportunity to improve the secondary prevention of cardiovascular and respiratory diseases through automated quantification of both CAC burden and emphysema extent.*

## Introduction

Lung cancer (LC), chronic obstructive pulmonary disease (COPD), and cardiovascular disease (CVD)—“the Big-3”—are expected to be the leading causes of death by 2050 globally [1]. Besides pulmonary nodules, low-dose computed tomography (LDCT) of the chest allows detection and quantification of coronary artery calcification (CAC) and pulmonary emphysema, recognized biomarkers for CVD and COPD, respectively [2, 3].

Driven by the evidence of a 20–40% reduction in LC mortality [4, 5], several national stakeholders and international scientific societies have been increasingly endorsing LDCT-based lung cancer screening (LCS) programs [6–9]. With the Big-3 sharing their main risk factors [1], these efforts toward LCS implementation might offer a unique opportunity to improve CVD and COPD secondary prevention through a large availability of LDCT images. Such a potentially huge number and heterogeneity of imaging data pose the need for a timesaving and reproducible approach for imaging analysis. The radiological literature explored the field of quantitative imaging, leading to the availability of objective parameters for assessing both emphysema and CAC, mostly exploiting Artificial Intelligence (AI)–based systems [10–12]. Nonetheless, whether and to what extent an objective and reproducible AI-based quantification of CAC and emphysema is affected by CT radiation dose is still to be defined.

This retrospective study aimed at assessing the consistency of automated measurements of CAC score and emphysema extent on CT images acquired with two different radiation dose protocols, namely LDCT and ultra-low-dose CT (ULDCT), using a commercially available AI software.

## Materials and methods

### Study population

**Fig. 1:**
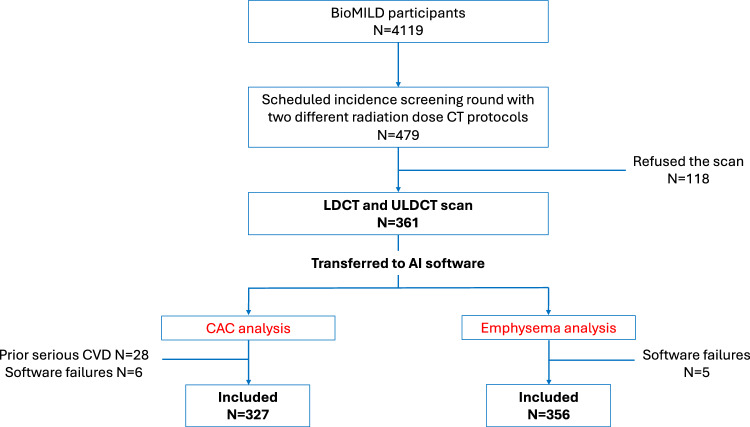
Flowchart of patient selection

This retrospective study was performed on prospective data acquired from the bioMILD trial (clinicaltrials.gov ID: NCT02247453), an ongoing prospective study testing the combination of plasma miRNA and LDCT to improve the efficacy of LCS by individual risk profiling and personalized screening intervals. Details on the bioMILD trial have been described elsewhere [13]. Of the 4119 bioMILD subjects (enrolled between January 2013 and March 2016), 479 consecutive volunteers scheduled to be recalled for an incident round between February and July 2019 were invited to be scanned with two different radiation dose CT protocols, as previously detailed [14]. Since 118 refused to participate, the patient cohort comprised 361 screenees (Fig. 1).

The original Institutional Review Board approval and written informed consent allowed the use of data for future research.

#### Imaging acquisition and reconstruction

LDCT scans were obtained with a third-generation dual-source CT scanner (Somatom Force, Siemens Healthineers); each participant underwent a double scan within the same single breath-hold: one LDCT scan (120 kVp and 25 mAs without tube-current modulation), acquired in the cranio-caudal direction, and one ULDCT, acquired in the caudo-cranial direction. ULDCT were acquired using a tin filter for spectral shaping and four different protocols:

1. ULDCT_1_: fully automated approach for modulation of tube potential (CARE kV, Sn100 kVp–Sn150 kVp) and automated exposure control (AEC for current at reference 100 mAs) on 92 subjects;
2. ULDCT_2_: fixed tube-voltage and tube current according to patient size (Sn100 kVp; 140 mAs for screenees whose field of view was 300 mm, 210 mAs for screenees whose FOV was 350/400 mm) on 91 subjects;
3. ULDCT_3_: hybrid approach with fixed tube-voltage at Sn100 kVp and AEC for current at reference 100 mAs on 85 subjects;
4. ULDCT_4_: hybrid approach with fixed tube-voltage at Sn150 kVp and AEC for current at reference 20 mAs on 93 subjects.

LDCT and ULDCT were reconstructed using a slice thickness of 1 mm, with an increment of 0.7 mm. A medium-sharp kernel (Br49) and advanced modeled iterative reconstruction (IR, ADMIRE strength level 3) were applied to LDCT scans, whereas two medium-sharp kernels and IR strength levels, namely Br49 (ADMIRE 3) and Qr49 (ADMIRE 4), were applied to ULDCT scans.

A more detailed description has been reported elsewhere [14].

### Clinical data

Data on past medical history, including data on cardiovascular risk factors, were collected through dedicated questionnaires and direct interviews with a study investigator at the baseline screening round.

### Coronary artery calcification and emphysema quantification

LDCT and ULDCT images were transferred to a dedicated workstation (Alienware Area 51 R6 equipped with Dual NVIDIA GeForce RTX 2080 OC graphics) and analyzed by a commercially available fully automated AI software (AVIEW, Coreline Soft; www.corelinesoft.com). As reported elsewhere [10], CAC was measured with a 3-dimensional U-net architecture-based scoring tool and stratified using three predefined Agatston score categories: 0–99, 100–399, and ≥ 400 [15]. Emphysema was quantified using the percentage of lung volume occupied by voxels with attenuation ≤ −950 Hounsfield Units, HU (percentage of low attenuation areas, %LAA) [16, 17]. %LAA values were stratified using three prespecified risk categories: ≤ 1, 1–5 and > 5 [18]. %LAA and CAC were computed after convolutional neural network-based sharp to soft tissue kernel conversion [19]. The kernel conversion algorithm was designed to obtain images mimicking standard-dose, low-frequency kernel images from low-dose, high-frequency kernel images [20].

### Statistical analysis

The study objective was the intra-subject comparison of CAC and %LAA values obtained with two different radiation dose CT protocols. Descriptive statistics were reported as numbers and percentages for categorical variables and as medians and interquartile ranges (IQR) for continuous variables. Agreement for CAC and %LAA categories was measured by the k-Cohen Index with Fleiss-Cohen weights (K_w_) and Intraclass Correlation Coefficient (ICC) with 95% Confidence Interval (CI). Correlations of continuous measurements were displayed by scatter plots and Bland Altman plots and tested by Pearson’s Correlation Coefficients.

Analyses were performed with Statistical Analysis System Software (SAS Studio 3.8, SAS Institute Inc.).

## Results

### Coronary artery calcification

Twenty-four volunteers were excluded due to previous history of CVD (i.e., myocardial infarction, thrombosis, stroke, and angina), four due to previous history of coronary stenting and six due to software failure in CAC measurements. The final cohort for CAC comparison analysis included 327 volunteers tested with LDCT and ULDCT protocols (Table 1).

**Table 1:** Comparison of CAC scoring

			ULDCT protocol CAC score			
			0–99	100–399	≥ 400	Median (IQR)
Total volunteers		327	219 (67.0%)	61 (18.7%)	47 (14.4%)	27.3 (2.4–158.8)
LDCT protocol CAC score	0–99	187 (57.2%)	186 (99.5%)	1 (0.5%)	0	3.7 (0.4–15.7)
	100–399	74 (22.6%)	32 (43.2%)	42 (56.8%)	0	107.5 (77.0–158.8)
	≥ 400	66 (20.2%)	1 (1.5%)	18 (27.3%)	47 (71.2%)	589.6 (351.5–1265.2)
	Median (IQR)	53.8 (1.6–303.2)	8.3 (0.2–54.8)	315.0 (195.5–444.1)	1089.4 (813.8–2241.2)	

Median value of CAC score on LDCT scan was 53.8 (IQR 1.6–303.2); 187 volunteers (57.2%) scored 0–99, 74 (22.6%) 100–399, and 66 (20.2%) ≥ 400.

Median value of CAC score on ULDCT scan was 27.3 (IQR 2.4–158.8); 219 volunteers (67.0%) scored 0–99, 61 (18.7%) 100–399, and 47 (14.4%) ≥ 400.

**Fig. 2:**
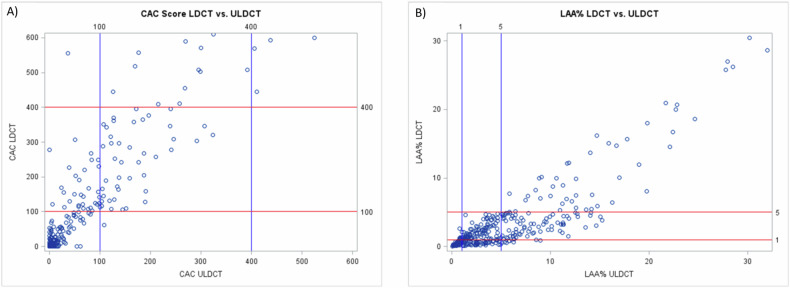
Correlation of CAC score (**A**) and emphysema extent (**B**) assessed on LDCT and ULDCT images

An overlap of CAC strata between LDCT and ULDCT scans was observed in 275/327 (84%) volunteers, with an almost perfect agreement: K_w_ of 0.86 (95% CI 0.82–0.90) and ICC of 0.86 (95% CI 0.79–0.90). LDCT and ULDCT CAC scores had a strongly positive correlation with Pearson’s Coefficient of 0.96 (*p* < 0.001), as shown in Fig. 2A (see also Supplementary Fig. 1).

### Emphysema

Five screenees were excluded due to software failure in %LAA measurements. The final cohort for %LAA comparison analysis included 356 volunteers (Table 2).

**Table 2:** Comparison of %LAA measurements

			ULDCT protocol %LAA			
			%LAA ≤ 1	%LAA 1–5	%LAA > 5	Median (IQR)
Total volunteers		356	67 (18.8%)	157 (44.15)	132 (37.1%)	3.5 (1.4–7.2)
LDCT protocol %LAA	%LAA ≤ 1	132 (37.1%)	62 (47.0%)	62 (47.0%)	8 (6.1%)	1.0 (0.5–2.9)
	%LAA 1–5	177 (49.7%)	5 (2.8%)	95 (53.7%)	77 (43.5%)	4.3 (2.4–7.0)
	%LAA > 5	47 (13.2%)	0	0	47 (100%)	14.0 (10.6–19.8)
	Median (IQR)	1.5 (0.6–3.8)	0.3 (0.2–0.7)	1.3 (0.7–2.3)	4.3 (2.4–7.4)	

Median value of %LAA measured on LDCT was 1.5 (IQR 0.6–3.8); 132 volunteers (37.1%) scored ≤ 1%, 177 (49.7%) 1–5%, and 47 (13.2%) > 5%.

Median value of %LAA measured on ULDCT was 3.5 (IQR 1.4–7.2); 67 volunteers (18.8%) scored ≤ 1%, 157 (44.1%) 1–5%, and 132 (37.1%) > 5%.

An overlap of %LAA strata between LDCT and ULDCT scans was found in 204/356 (57%) volunteers, with a moderate agreement: K_w_ of 0.57 (95% CI 0.51–0.63) and ICC of 0.57 (95% CI 0.21–0.75). LDCT and ULDCT %LAA had a strongly positive correlation with Pearson’s Coefficient of 0.876 (*p* < 0.001), as shown in Fig. 2B (see also Supplementary Fig. 2).

### Radiation exposure

Dose-length product (DLP) values were lower for ULDCT than LDCT, with the ULDCT_3_ protocol showing the lowest values of both volume CT dose index (CTDIvol) and effective dose (ED). Detailed data are reported in Table 3.

**Table 3:** Radiation exposure of subjects for LDCT and ULDCT scans

		Group ULDCT_1_	Group ULDCT_2_	Group ULDCT_3_	Group ULDCT_4_
		92 subjects	91 subjects	85 subjects	93 subjects
DLP (mGycm)	LDCT	63.9 (60.6–68.9)	64.7 (60.5–68.8)	65.0 (62.3–70.5)	64.4 (61.1–68.1)
	ULDCT	22.0 (17.3–28.3)	25.8 (23.0–35.6)	19.2 (16.3–23.8)	26.3 (22.0–30.2)
CTDIvol (mGy)	ULDCT	0.55 (0.44–0.70)	0.59 (0.59–0.89)	0.47 (0.40–0.58)	0.64 (0.56–0.74)
ED (mSv)		0.31	0.36	0.27	0.37

*CTDIvol* volume CT dose index, *ED* effective dose, *DLP* dose-length product, *LDCT* low-dose computed tomography, *mGy* milligray, *mSv* millisievert, *ULDCT* ultra-low-dose computed tomography

## Discussion

We tested the feasibility of automated CAC and emphysema quantification on ULDCT scans of subjects participating in a LCS program by comparing these results with those from the LDCT scans acquired within the same breath-hold. We observed an overlap of CAC risk categories in 84% of screenees with an almost perfect agreement, while the overlap for %LAA categories was overall lower, showing only a moderate agreement. These results are of particular interest given the burgeoning number of sites planning to start or already performing LCS activities. The reproducibility of measurements for CAC and emphysema intrinsically favors the applicability of LCS within national health systems, where different CT scanners and acquisition protocols are expected.

Although the agreement was almost perfect (K_w_ of 0.86), a tendency towards a CAC score underestimation of ULDCT imaging was observed for 100–399 and ≥ 400 categories, whereas an opposite trend was noticed for the lowest risk category. Previous literature reported lower specificity and sensitivity of ULDCT imaging as compared to standard-dose CT in this setting, but the potential improvement of results due to the automated approach was not explored [21]. Gorenstein et al recently tested a novel AI-based reconstruction denoising method for assessing both pulmonary nodules and CAC on ULDCT imaging in a LCS cohort of 123 subjects [22], demonstrating an accuracy of ULDCT imaging of 91.7% for CAC assessment.

Albeit through different software and metrics, previous studies compared the detection of emphysema on ULDCT and LDCT imaging, demonstrating that visual assessment might be particularly challenging on ULDCT images with variable sensitivities reported [23]. Wang et al observed that iterative reconstruction (IR) algorithms improve the accuracy of ULDCT, reducing the overestimation of subtle emphysema when compared to ULDCT scans reconstructed with analytic reconstruction algorithms, namely filtered back projection [24]. Kim et al tested two different ULDCT protocols (i.e., 100 kVp/20 mAs and 80 kVp/30 mAs) using IR in a non-LCS population, reporting that very low radiation doses should be avoided when emphysema is suspected [25]. The latter study, however, included a rather small number of subjects (25, of whom only 6 with emphysema), somehow limiting the significance of the observations. It is worth underlining that the acquisition was performed during the same breath-hold, thus excluding potential variations caused by different inspiration levels of physiological changes within different time points. Moreover, images were reconstructed with an IR algorithm (ADMIRE), which was demonstrated to ensure higher image quality in terms of contrast-to-noise ratio (CNR) than that used by Kim et al (SAFIRE) [26].

Notably, some Authors have suggested that thresholds higher than −950 HU (e.g., −940 HU, −930 HU) should be preferred for quantifying emphysema at LDCT imaging [27, 28]. As such, it can be speculated that a simple adjustment of software thresholds for emphysema quantification might overcome the degree of overestimation that we observed with ULDCT protocols.

Our preliminary results seem to indicate that a fully automated approach for CAC and emphysema quantification might be feasible in the setting of LCS, with more than 90% of disagreeing strata classification falling into the nearest risk category (Tables 1 and 2). The differences observed between the two protocols, however, suggest that a fully automated assessment should be critically interpreted when referring individuals for clinical evaluation and that the use of a standardized acquisition protocol should be encouraged in the setting of LCS, to improve the measuring reproducibility.

This study has some limitations. First, as inherent to the nature of any retrospective study, our findings are subject to confounding factors. Second, the monocentric design with a single CT vendor reduces the generalizability of our results. Third, we only assessed the consistency of CAC score and %LAA categories between two different radiation dose CT protocols, but we did not test the diagnostic performance of ULDCT towards LDCT.

In conclusion, CAC quantification performed by a fully automated AI software showed similar results when applied on CT images acquired with different radiation dose CT protocols in a LCS population, while the quantification of emphysema tended to be overestimated on ultra-low-dose CT protocols.

## Supplementary information

ELECTRONIC SUPPLEMENTARY MATERIAL

## Abbreviations

AEC: Automated exposure control
AI: Artificial intelligence
CAC: Coronary artery calcification
CI: Confidence interval
COPD: Chronic obstructive pulmonary diseases
CTDIvol: CT dose index
CVD: Cardiovascular disease
DLP: Dose-length product
ED: Effective dose
HU: Hounsfield Units
ICC: Intraclass correlation
IQR: Interquartile ranges
IR: Iterative reconstruction
LAA: Low attenuation areas
LDCT: Low dose computed tomography
LC: Lung cancer
LCS: Lung cancer screening
ULDCT: Ultra-low-dose computed tomography
