# Supplementary material for: Coronary calcium score and emphysema extent on different CT radiation dose protocols in lung cancer screening
Source: Eur Radiol. 2024 Dec 20;35(7):3781–7. doi: 10.1007/s00330-024-11254-w (PMC12165900; doi:10.1007/s00330-024-11254-w)

Coronary calcium score and emphysema extent on different CT radiation dose protocols in lung cancer screening

ELECTRONIC SUPPLEMENTARY MATERIAL

Supplementary figure 1. Bland-Altman plot of LDCT and ULDCT CAC scores.

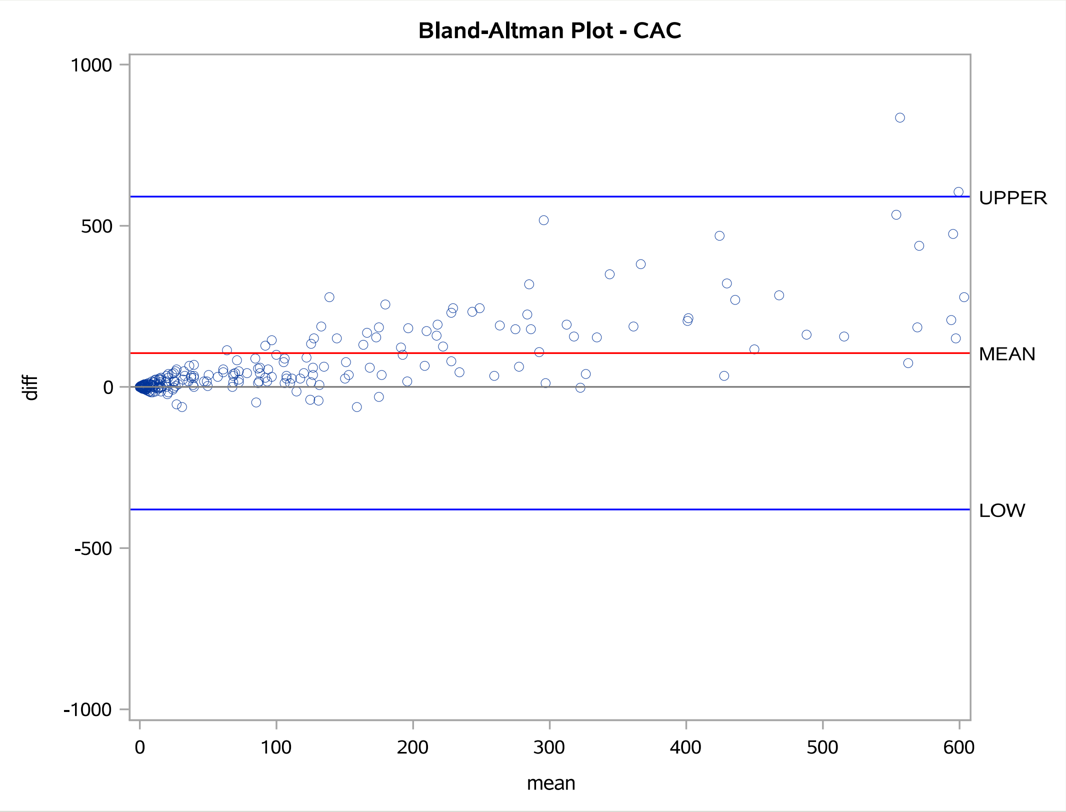

Supplementary figure 2. Bland-Altman plot of LDCT and ULDCT LAA% measurements.

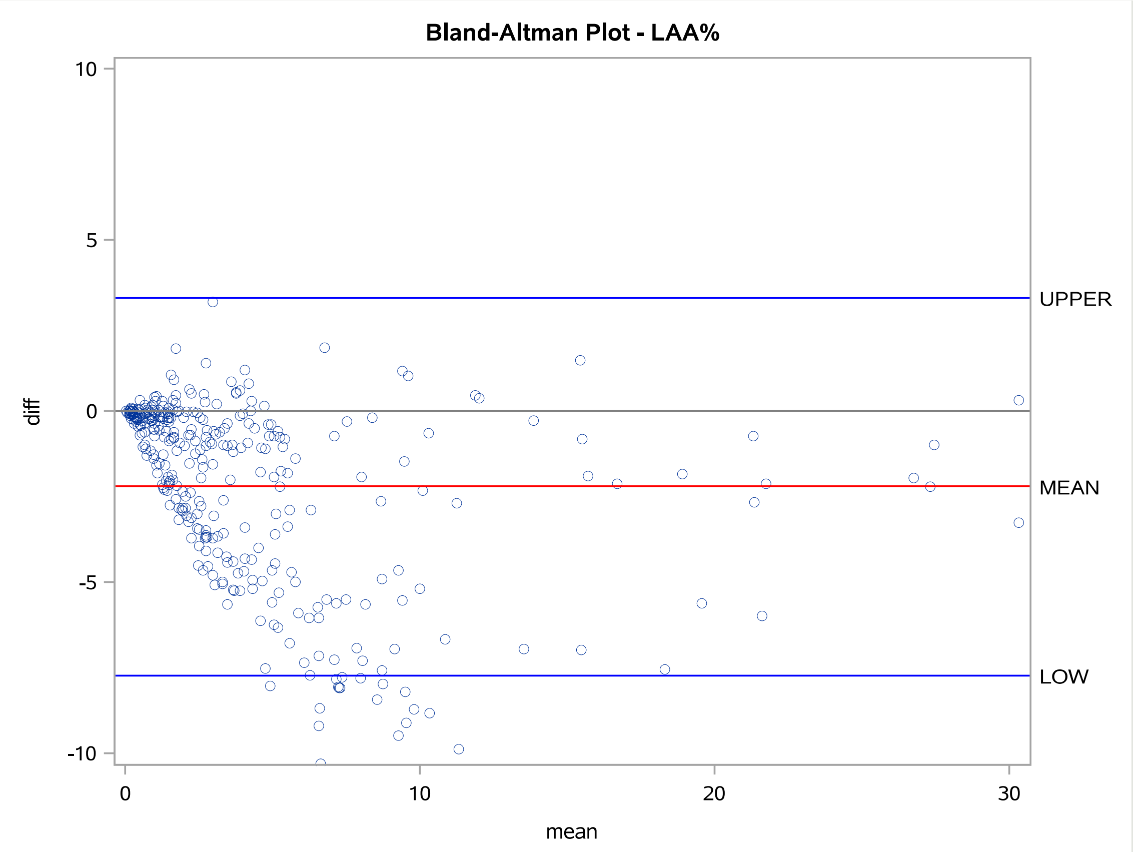

Supplement: Supplementary file 1 — ELECTRONIC SUPPLEMENTARY MATERIAL [file 330_2024_11254_MOESM1_ESM.pdf]
